# Centering Equity and Developing the Maternal Health Workforce: Building the National Maternal Health Learning and Innovation Center

**DOI:** 10.1007/s10995-022-03382-w

**Published:** 2022-03-18

**Authors:** Julia Reddy, Amy Mullenix, Abby C. Cannon, Deitre Epps, Christine Tucker

**Affiliations:** 1 Center of Excellence Trainee, Department of Maternal and Child Health, University of North Carolina at Chapel Hill, 412 Rosenau Hall, Chapel Hill, NC 27599 USA; 2 Maternal Health Learning and Innovation Center, Department of Maternal and Child Health, University of North Carolina at Chapel Hill, 412 Rosenau Hall, Chapel Hill, NC 27599 USA

**Keywords:** Workforce development, Maternal health, Racial equity, Training and technical assistance

## Abstract

**Purpose:**

The purpose of this article is to describe the development of the Maternal Health Learning and Innovation Center (MHLIC), a national initiative designed to enhance workforce capacity of maternal health professionals in the United States.

**Description:**

The mission of the MHLIC is to foster collaboration and learning among diverse stakeholders to accelerate evidence-informed approaches advancing equitable maternal health outcomes through engagement, innovation, and policy. Working to center equity in all efforts, the MHLIC builds workforce capacity through partnership, training, technical assistance, coaching, facilitation of peer learning, and a national resource repository.

**Assessment:**

The MHLIC employed several assessment strategies in its first year, including a baseline learning survey of awardees, a stakeholder survey of potential collaborators in maternal health, and advisory convenings. Internally the MHLIC team assessed its own intercultural development. Assessment results informed internal and external approaches to workforce development.

**Conclusions:**

Telehealth implementation, access to services for rural populations, racial inequities, and data use and dissemination were the primary gaps that awardees and other stakeholders identified. The MHLIC is unique in its collaborative design approach and the centering of equity as foundational to the structure, subject, and culture of its work. The MHLIC utilizes a collaborative approach that capitalizes on academic and practice partners’ extensive expertise in maternal health systems. Key to the success of future maternal health efforts is workforce development that builds the awareness and capacity to advance racial and geographic equity for public health, community, and clinical professionals.

## Significance Statement

*What is already known about the topic?* Workforce development for maternal health (MH) practitioners has been shown to effectively enhance the capacity of the MH workforce and advance outcomes. Federal funders recognized a national need for a centralized hub to build the capacity of MH practitioners and to serve as a repository for information and best practices that speed translation of effective strategies into practice.

*What this article adds?* This description of the MHLIC model informs other capacity building and resource gathering endeavors by describing the process of developing and operationalizing equity as the cornerstone of maternal health workforce development.

## Purpose

The United States is the only advanced economy in which the maternal mortality rate is increasing (GBD 2015, 2016). Health disparities associated with systemic racism manifest in maternal mortality and morbidity, with Black women having significantly higher risks, even after adjusting for an array of other demographic characteristics (Howell, 2018; Petersen et al., 2019). The Health Resources and Services Administration (HRSA), the Centers for Disease Control and Prevention (CDC), and the American College of Obstetricians and Gynecologists (ACOG) invest in innovative projects to reverse this trend, including the Alliance for Innovation on Maternal Health (AIM) maternal safety bundles, which represent best practices for maternity care (Mahoney, 2018). However, efforts to affect the national incidence of maternal mortality and severe maternal morbidity that target clinical care often fail to address the skills and capacity of the non-clinical workforce. In addition, variable legislative agendas, short grant timelines, and siloed data collection complicate efforts to capitalize upon gains on a national scale (Collier & Molina, 2019).

Workforce development for maternal and child health practitioners has been shown to effectively enhance capacity and advance innovation (Clarke & Cilenti, 2018; Fleming et al., 2019). National maternal health (MH) organizations like Black Mamas Matter Alliance, National Birth Equity Collaborative, Every Mother Counts, the 4th Trimester Project, and the National Perinatal Task Force have improved the field’s understanding of racial, ethnic, and geographic disparities in adverse outcomes. These recognize an important shift away from simply describing disparities and toward actively centering equity in MH initiatives (Howell et al., 2018). To shift towards centering equity, training and resources must include emphases on inclusion of lived experience, strong community partnerships, a direct accounting for structural racism, and information and data that centers racial equity (Hawn Nelson et al., 2020; Muse et al., 2018).

This article describes the development of the Maternal Health Learning and Innovation Center (MHLIC), a national initiative designed to enhance workforce capacity of MH professionals in the United States. As this manuscript outlines the organizational processes of developing a national technical assistance (TA) center and all data come from program evaluation results, it was deemed as non-human subjects research by the UNC Institutional Review Board.

## Description

**Fig. 1:**
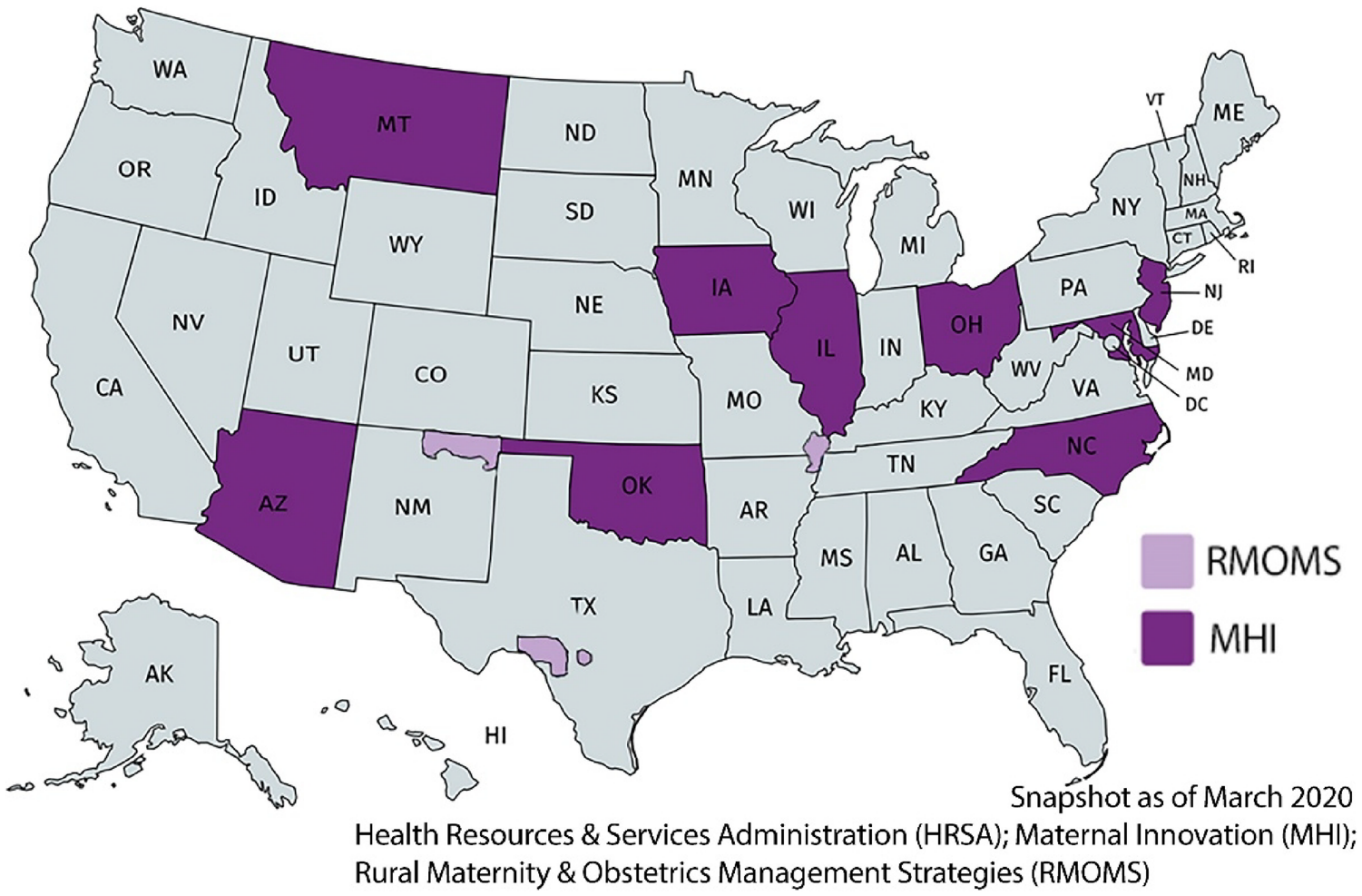
The nine Health Resources and Services Administration (HRSA) funded State Maternal Health Innovation (MHI) and three Rural Maternity & Obstetrics Management Strategies (RMOMS) awardees

**Fig. 2:**
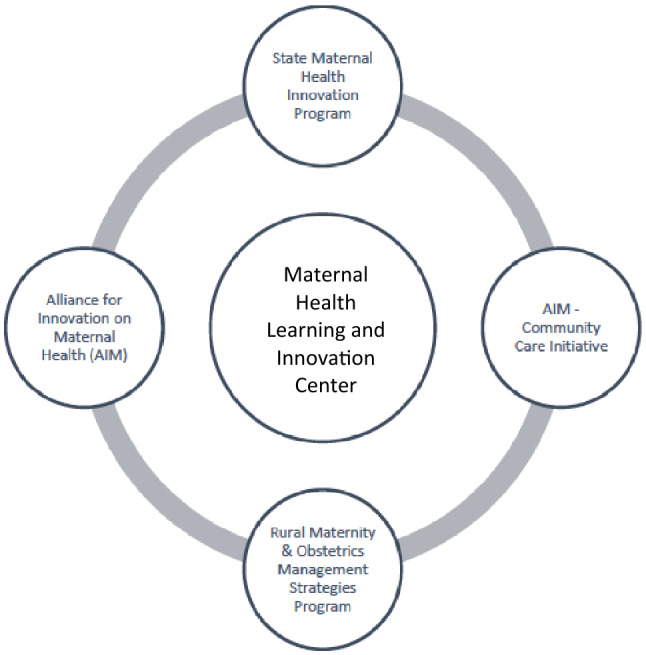
The Health Resources and Services Administration (HRSA) Maternal Health Programming Organizational Chart, including the Maternal Health Learning and Innovation Center, which is the funded by the Supporting Maternal Health Innovation Program

In October 2019, the MHLIC was launched in conjunction with the funding of nine maternal health innovation (MHI) state-level initiatives, based primarily within Title V programs, and three Rural Maternity and Obstetrics Management Strategies (RMOMS) programs (Fig. 1). The MHLIC, housed at UNC-Chapel Hill, supports these twelve award recipients (referred to in this article as “awardees”) and serves as a capacity-building resource center and repository of innovation strategies for MH professionals nationally including AIM awardees (Fig. 2).

The mission of the MHLIC is to foster collaboration and learning among diverse stakeholders to accelerate evidence-informed interventions advancing equitable MH outcomes through engagement, innovation, and policy. The MHLIC takes a developmental approach to growing its own intercultural development so that it can better support MH practitioners in their MH equity work. Underlying the creation of the center was an iterative process of collaboration in design, which included a Baseline Learning Survey, administered to awardee members. The results of this survey, and on-going input and evaluation, inform the evolving TA offerings of the MHLIC.

### Inclusive Partnership

Dedicated partnership is central to the structural design of the MHLIC and advances the goal of centering equity and making a broad range of expertise available to the MH field. UNC convened a multidisciplinary team and houses the MHLIC hub, which includes the evaluation, training and communications teams and the Core Manager, Program Awardee Liaison, and the Senior Collaboration Manager. Working in partnership with three schools at UNC (public health, social work, and medicine), six additional organizations provide a range of educational and advocacy expertise and diverse perspectives to facilitate the provision of comprehensive TA and workforce development. Prior to applying for funding, UNC built relationships with organizations with expertise in addressing MH inequities and concurrent expertise in training, TA, and capacity building. One additional academic partner and five practice-based partners signed on to become MHLIC partners. The Georgia Health Policy Center specializes in research translation and TA; Reaching Our Sisters Everywhere (ROSE), a Georgia-based non-profit, focuses on culturally competent training, education, advocacy, and breastfeeding support within African American communities; R.A.C.E. for Equity, a global consulting firm, provides culturally-relevant TA and training for data-driven decision making, equity, and intercultural development; ACOG, a professional membership organization for obstetricians and gynecologists, coordinates alignment between AIM and MHLIC; the Association of Maternal and Child Health Programs liaises with state and organizational public health leaders; and Ph Solutions serves as the Program Awardee Liaison and oversees implementation coaching.

**Fig. 3:**
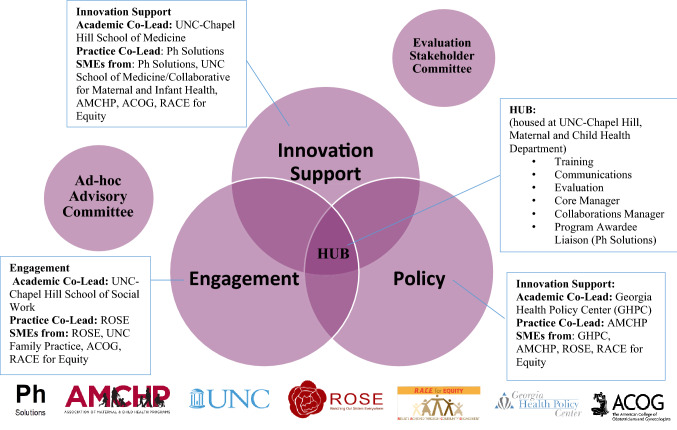
The MHLIC is organized in three “cores”, each co-led by an academic partner and a practice partner and include Subject Matter Experts (SMEs) from partner organizations

The MHLIC fosters cross-pollination among academic and practice-based partners in workgroups, called cores, that center around three areas: Engagement, Innovation Support, and Policy (Fig. 3). Each core is co-led by a practice-based and an academic partner. Subject matter experts (SMEs) from each partner organization attend core meetings and work with members of the UNC-hub to build workforce development resources. An equity expert participates on each core to ensure attention to equity-informed practice and understanding the community perspective. In addition, the MHLIC assembled ad-hoc advisory and evaluation stakeholder committees, comprised of multi-disciplinary partners, including awardees, to inform and advise the MHLIC.

### Training, TA, and Coaching

The MHLIC manages a robust training program, co-creating curricula that strive to be equity-oriented, responsive, timely, and engaging. This includes online learning resources, National Symposia, and other targeted subject matter training opportunities, including Communities of Practice (Table 1). The MHLIC offers TA to all awardees and other interested stakeholders by request. The TA process begins with exploration of needs and goals, followed by connections to MHLIC subject matter experts and other resources.

**Table 1:** Types of support the Maternal Health Learning Innovation Center provides and the audience served

Type of support	Description of support	Intended audience
Inclusive partnership	Convene an ad-hoc advisory committee and evaluation stakeholder committee, attend regular meetings with maternal health partners including AIM Community Care Initiative’s Maternal Safety Workgroup and Centers for Disease Control Maternal Mortality Prevention Team, connect awardees to one another and to local and national partners	Maternal Health and Technical Assistance Partners
Training	Develop and deliver curricula through coordinated activities to increase the knowledge, skills, and abilities of clinical and non-clinical service providers	All maternal health practitioners
Technical assistance	Provide and/or facilitate culturally relevant and expert programmatic, scientific, and technical advice	All maternal health practitioners
Coaching	Technical advice and support	State MHI and RMOMS awardees
Peer learning opportunities	Convene community of learners in communities of practice; host learning institute and national symposium	All maternal health practitioners and more intensively for state MHI and RMOMS awardees
Resource dissemination	Identify existing resources and produce new resources. Share all resources through an accessible online repository: maternalhealthlearning.org	All maternal health practitioners

Each awardee communicates directly with a designated coach and the MHLIC Program Awardee Liaison, both of whom offer resources and connections to subject matter and capacity-building experts. Implementation coaching has been documented as an effective mechanism for translation of innovations into practice (Meyers et al., 2012). Coaches work with teams to identify and address capacity gaps and implementation challenges, and to broker on-going support and resources. Coaches facilitate connections between awardee teams and connect awardees with resources.

### Peer Learning

A key element of training and supporting MH professionals is fostering a sense of cohesion and peer-to-peer support (Brandt et al., 2018). The MHLIC has built a community of peer learners among awardees to enhance the effectiveness of implementation activities and support movement from learning to action. The community of peer learners connect virtually, have access to regular learning webinars, and present their work at Community of Practice meetings and larger MHLIC convenings.

### National Resource Center

Research suggests that more than a decade can pass between establishment of best practice or promising innovation and wide political or clinical adoption (Morris et al., 2011). Non-clinical MH professionals, such as those in state Title V agencies, lack access to academic literature or other emergent resources offering best practice guidance. In response, the MHLIC launched the Resource Center section of its website (maternalhealthlearning.org) in January 2021. The site is a comprehensive and well-vetted source for all MH professionals to enhance skills and knowledge. MH professionals request TA via this website, which also houses trainings, recorded webinars, and other learning resources for both clinical and public-health oriented practitioners.

### Centering Equity

Woven throughout the design, internal functioning, and operations of the MHLIC is a commitment to racial and geographic equity. The internal equity work is led by R.A.C.E. for Equity and began by advising on hiring practices of the UNC-hub team and by building a common language, defining terms such as diversity, equity, and inclusion, and developing mission and vision statements. Recognizing UNC as a predominantly white institution, a concerted effort was made to grow a diverse team and to hire people with lived experience navigating maternal health systems. MHLIC includes equity as a central part of each of the planning, process, and core workgroups, rather than having a separate equity work group. All members of the MHLIC team and partnership are expected to work towards advancing equity on an individual, team, and organizational level.

The MHLIC is a learning organization that focuses on continually expanding knowledge of and capacity to advance equity. The MHLIC team engages in transparent dialogue regarding cross-cultural issues. To assess baseline understanding among MHLIC team members, R.A.C.E. for Equity led an Intercultural Development Inventory ® (IDI) assessment (Hammer et al., 2003), during which 47 of the 49 MHLIC staff members self-assessed their intercultural development. Group and individual processing and coaching sessions were used to develop plans for individual and team growth. Regular equity caucuses offer unstructured opportunities for staff reflection and learning. Staff began sessions all together and then separated by racial and ethnic identities so that black indigenous and people of color (BIPoC) and non-BIPoC staff could process individually. Participation in racial equity training was also an important focus for MHLIC team members’ own professional development. These opportunities, along with ongoing self-assessment, equip MHLIC team members to better respond to training and TA requests and to model what the center is learning from its own equity work in their workforce development efforts.

## Assessment

The MHLIC employed several strategies to assess MH workforce development needs in the center’s first year. A stakeholder survey solicited priorities from a broad group of potential collaborators. Advisory and evaluation stakeholder committee meetings helped determine emerging best practices to meet the primary aims of the center. A baseline learning survey provided a roadmap for how to promote innovation and sustain workforce momentum.

### Stakeholder Survey

Respondents of the MH stakeholder survey (n = 32), representing state public health departments and national and grassroots organizations, identified the most common gaps in MH resources as: inclusion of rural/frontier needs; racial inequality and implicit bias; access to disaggregated data; barriers to telehealth; and maternal mental health. Respondents shared toolkits, trainings, curricula, and other resources for the resource repository. Feedback from respondents also guided MHLIC partnership development.

### Advisory Committees

A primary feature of the MHLIC structure is the collaborative design approach, where state partners and MH professionals co-create center offerings. MHLIC leadership held ad hoc advisory meetings with 21 state and regional professionals to determine strategies and emerging best practices for MH workforce development. Attendees contributed ideas on how to deliver virtual capacity-building trainings and how to center racial equity, and identified gaps in resources and support in existing coaching and training efforts. Resulting feedback guided the center’s foundational work and training and TA offerings. Additionally, an evaluation stakeholder committee (n = 24) was convened with representatives from national and community partners, government funders, and awardees to advise on proposed evaluation strategies.

### Baseline Learning Survey

The baseline learning survey of awardees identified workforce strengths and gaps, and informed training and TA efforts. Fifty-eight MH professionals (43 MHI and 15 RMOMS) across all twelve awardee teams completed the survey. Awardees reported that they wanted a formal structure to engage in peer learning and resource sharing (e.g. data collection templates), and guidance on topics such as implementing broad-scale innovations. Telehealth implementation was the most common knowledge gap identified. Other areas that awardees hoped to improve upon included addressing maternal mortality risk factors and racial disparities, advancing birth equity, better serving rural and frontier populations, and improving data use and dissemination.

## Results

Assessment results guided the development of training, TA, peer learning, and the resource repository in MHLIC’s first year. The MHLIC identified telehealth implementation, rural and frontier needs, how to address racial inequities, and data use and dissemination as primary knowledge gaps that awardees and other stakeholders wanted to address. As the MHLIC pivoted to virtual platforms due to the COVID-19 pandemic, training and TA topics were amended to address the impact of COVID-19, such as skills for conducting virtual convenings. At all points, the MHLIC attempted to elevate equity as a foundational component of its learning content.

Coaching and TA records provide detail on how the MHLIC met the workforce development needs of MH practitioners. Coaching began in January 2020 and the MHLIC logged 132 coaching interactions by September 2020. The most common coaching topics were formation of taskforces, evidence-informed strategies to improve outcomes, and maternal telehealth strategies. The most common capacity-building areas were effective partner engagement and equity.

In February 2020, the first TA request was received, with MHLIC completing 32 total requests by September 2020. Examples of TA included: state-level analyses of current maternal morbidity/mortality rates and relevant women's health policies to inform taskforce recommendations; identifying a screening tool for Social Determinants of Health to pilot in a two-generation clinic; and developing guidance on engaging stakeholders for meaningful support on a MH taskforce. Additionally, teams were connected with one another, for example, to explore whether a REDCap database used in one state could be replicated in another.

MHLIC is committed to ensuring that TA, trainings, and resources focus on centering equity. MHLIC hosted a webinar series on equity and engagement, compiled a list of racial equity trainings and resources for MH, hosted Learning Institute sessions on equity, and convened awardees to discuss implementation and evaluation of equity trainings. All external MHLIC convenings begin with a land acknowledgement. The Center regularly reviews internal work plans and deliverables with an eye toward centering equity and continues to build and promote the equity section of the resource repository.

## Conclusion

An academic-practice partnership and an equity focus were foundational to the creation of a nationally prominent resource repository and training/TA Center, which disseminates capacity-building tools to stakeholders and expedites the translation of knowledge to practice. As a mutual-learning organization, MHLIC leverages the expertise of partners, the funder (MCHB) and the larger MH community to inform development of new capacity-building resources to improve outcomes and eliminate inequities.

### Lessons Learned

Because of the complexity of maternal health inequities, the MHLIC was intentionally designed to include a diverse collaborative team made up of more practice-based than academic partners, including BIPoC-led organizations. All activities articulate the goal of an ongoing learning process in which equity is operationalized collectively and intentionally over time.

Specific lessons learned to date include:

- Invest in and prioritize capacity building for staff and partners to deepen their individual awareness and commitment to advancing health equity.
- Allocate time for a multi-partner initiative to settle on framing and definitions related to equity. Because of the diversity of perspectives in the MHLIC, this process is developmental and ongoing, building from interactions with awardees and MHLIC’s own internal equity learning.
- Ensure collaboration is attended to, both internally and externally. Communication must be direct and on-going to protect the unity and engagement of staff and the layers of partners and constituents.

The MHLIC is unique in its collaborative partnership design and the centering of equity as foundational to the structure, subject, and culture of its workforce development and capacity-building charge. As the demand for equity-informed MH practitioners grows, the MHLIC will continue to co-design equity-centered products in collaboration with awardees and other stakeholders. The MHLIC recognizes the importance of including people with lived experience in the design of capacity building resources and is committed to learning ways to do this well and to share what is learned along the way. Partnering with an equity expert and prominent practice organizations, investing in the IDI assessment and intercultural coaching as tools for growth, and making time for authentic conversations to develop a shared language, mission, and vision have been key in building equity into the design of the center and ultimately improving the quality and functioning of this national workforce development effort.
